# Supplementary figures and images for: Biologically-informed neural networks guide mechanistic modeling from sparse experimental data
Source: PLoS Comput Biol. 2020 Dec 1;16(12):e1008462. doi: 10.1371/journal.pcbi.1008462 (PMC7732115; doi:10.1371/journal.pcbi.1008462)

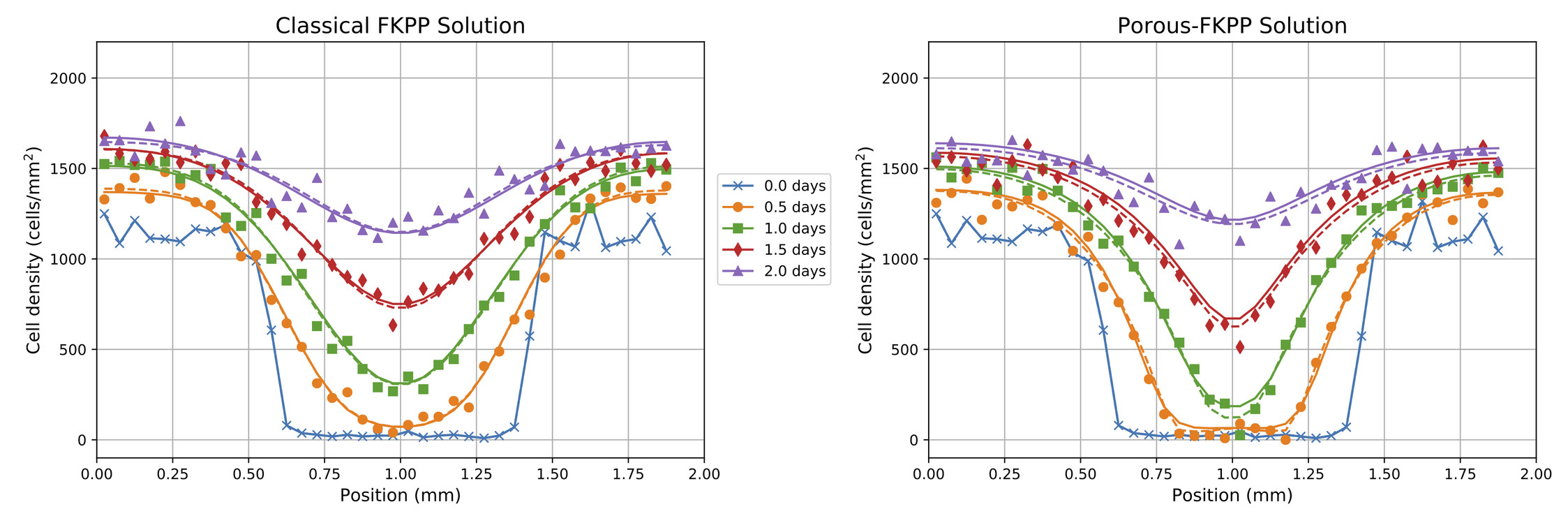

Supplement: S1 Fig — Predicted cell density profiles using BINNs with the governing reaction-diffusion PDE in Eq (9). The left subplot corresponds to the set of simulated data using the classical FKPP equation and the right subplot corresponds to the Generalized Porous-FKPP equation. Solid lines represent the numerical solution to Eq (9) using DMLP, and GMLP. Dashed lines represent the noiseless numerical simulations of the classical FKPP and Generalized Porous-FKPP equations. The markers represent the numerical simulations of the classical FKPP and Generalized Porous-FKPP equations with artificial noise generated by the statistical error model in Eq (4). (TIF) [file pcbi.1008462.s001.tif]

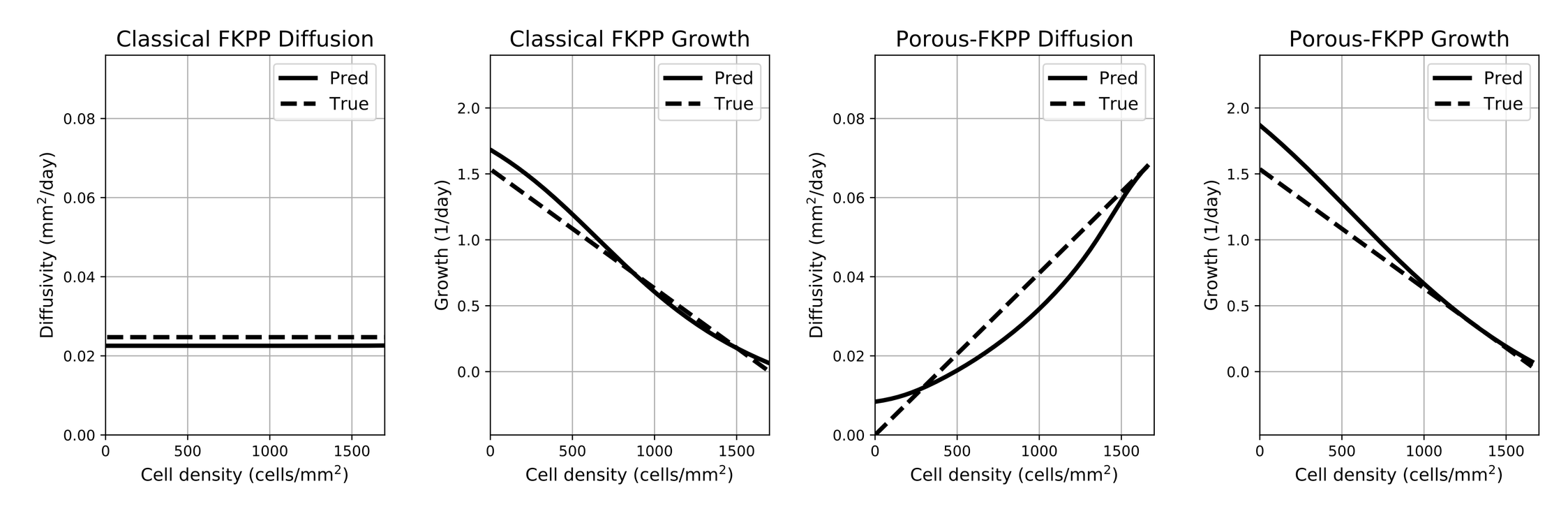

Supplement: S2 Fig — The learned diffusivity and growth functions DMLP and GMLP evaluated over cell density u. Starting from the left, the first two subplots correspond to the learned diffusivity and growth functions from simulated data using the classical FKPP equation. The last two subplots correspond to the learned diffusivity and growth functions from simulated data using the Generalized Porous-FKPP equation. Solid lines represent the parameter networks DMLP and GMLP and dashed lines represent the true diffusivity and growth functions used to simulate the data. (TIF) [file pcbi.1008462.s002.tif]

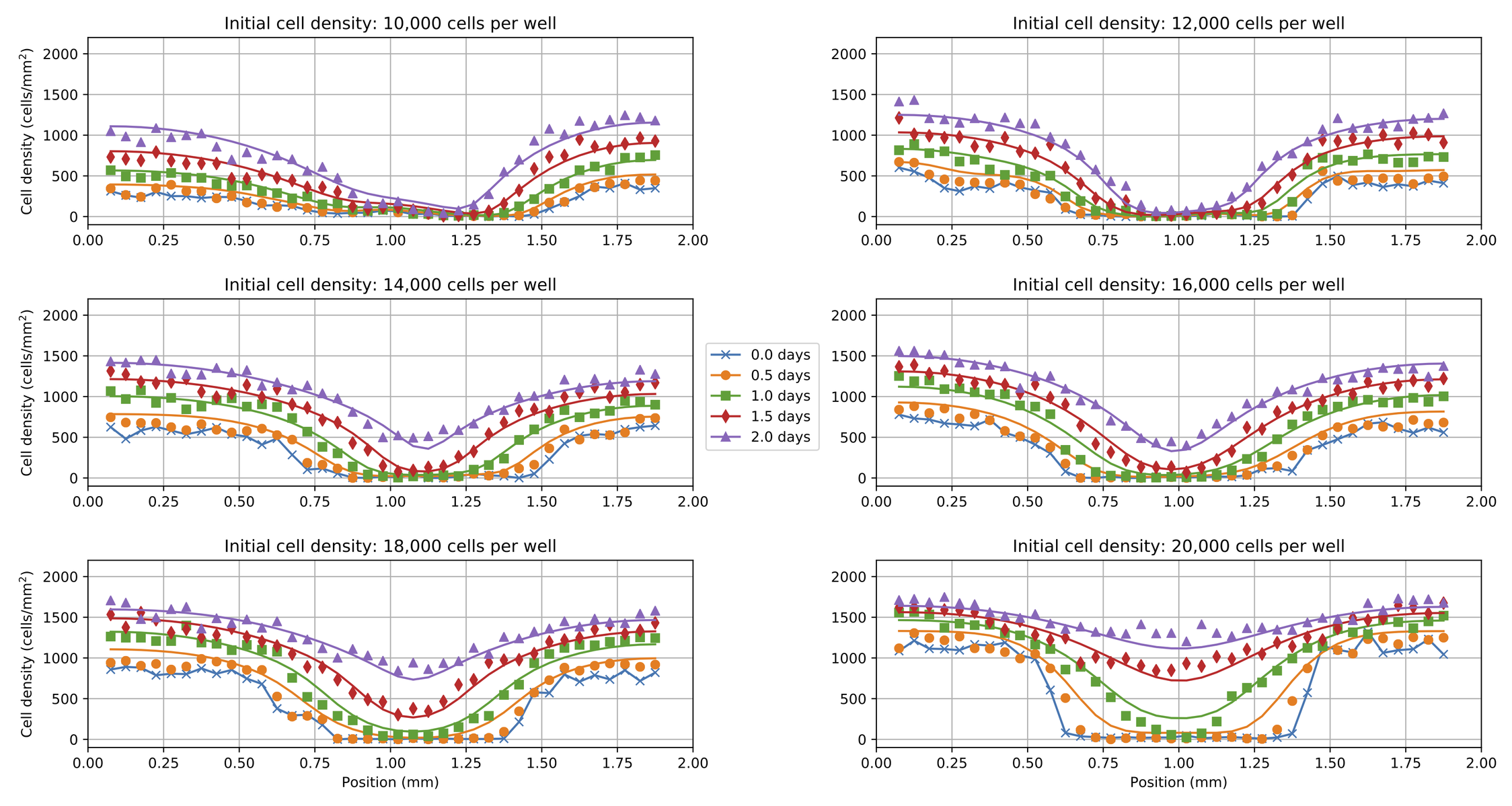

Supplement: S3 Fig — Predicted cell density profiles using BINNs with the governing reaction-diffusion PDE in Eq (9). Each subplot corresponds to an experiment with a different initial cell density (i.e. 10,000, 12,000, 14,000, 16,000, 18,000, and 20,000 cells per well). Solid lines represent the numerical solution to Eq (9) using DMLP and GMLP. The markers represent the experimental scratch assay data. (TIF) [file pcbi.1008462.s003.tif]

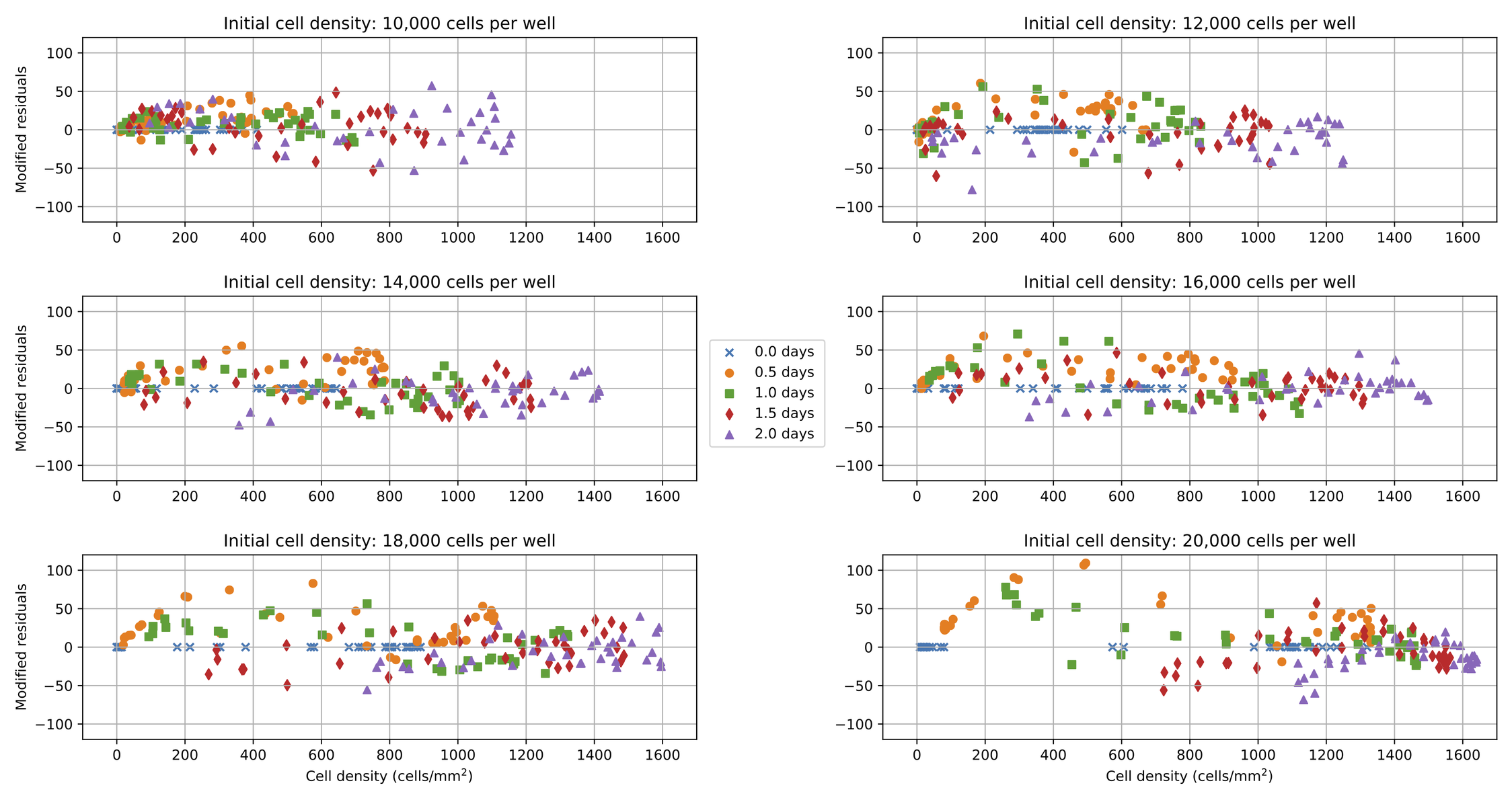

Supplement: S4 Fig — Modified residuals using BINNs with the governing reaction-diffusion PDE in Eq (9). Each subplot corresponds to an experiment with a different initial cell density (i.e. 10,000, 12,000, 14,000, 16,000, 18,000, and 20,000 cells per well). (TIF) [file pcbi.1008462.s004.tif]

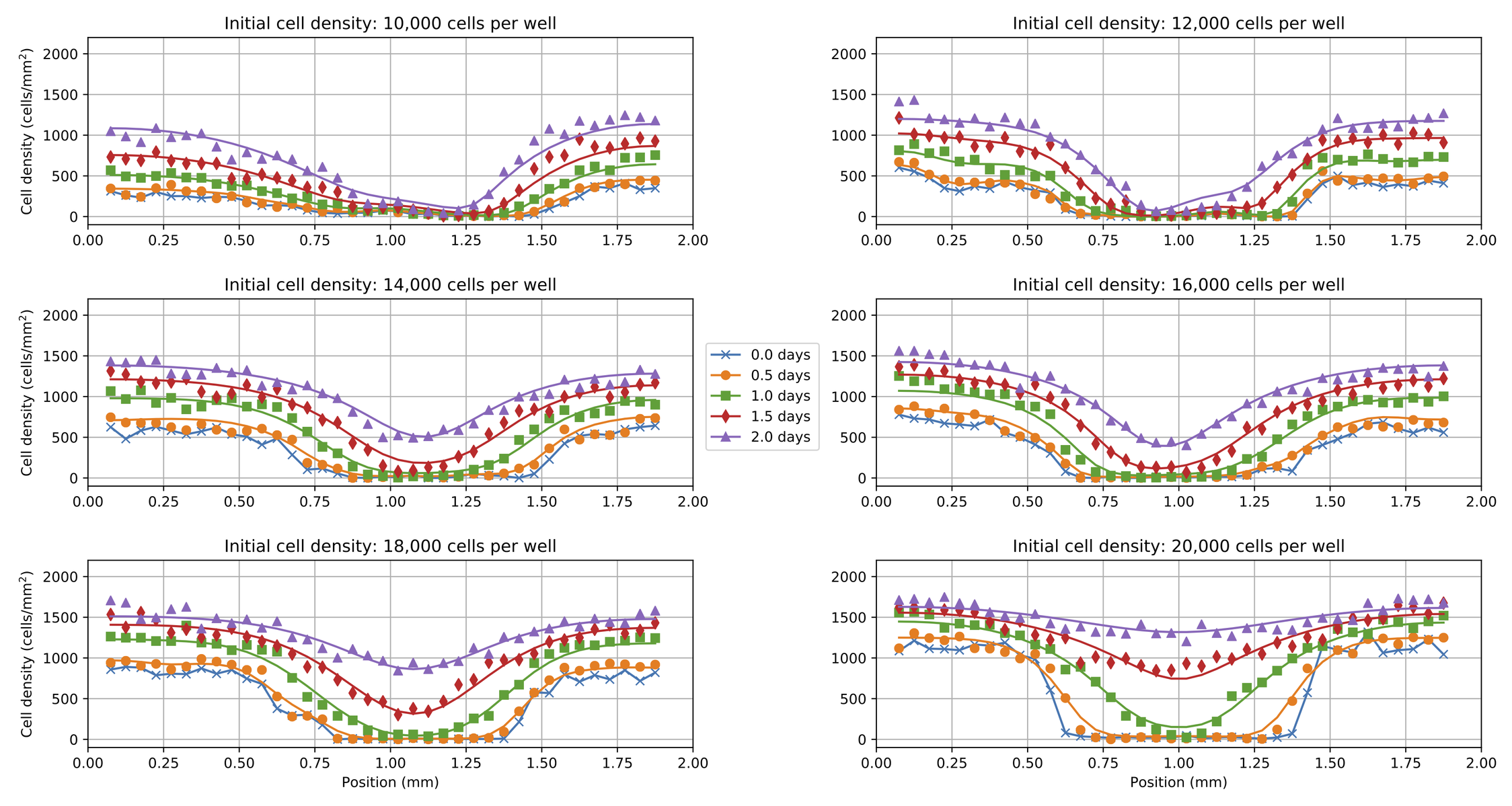

Supplement: S5 Fig — Predicted cell density profiles using BINNs with the governing delay-reaction-diffusion PDE in Eq (10). Each subplot corresponds to an experiment with a different initial cell density (i.e. 10,000, 12,000, 14,000, 16,000, 18,000, and 20,000 cells per well). Solid lines represent the numerical solution to Eq (10) using TMLP, DMLP, and GMLP. The markers represent the experimental scratch assay data. (TIF) [file pcbi.1008462.s005.tif]

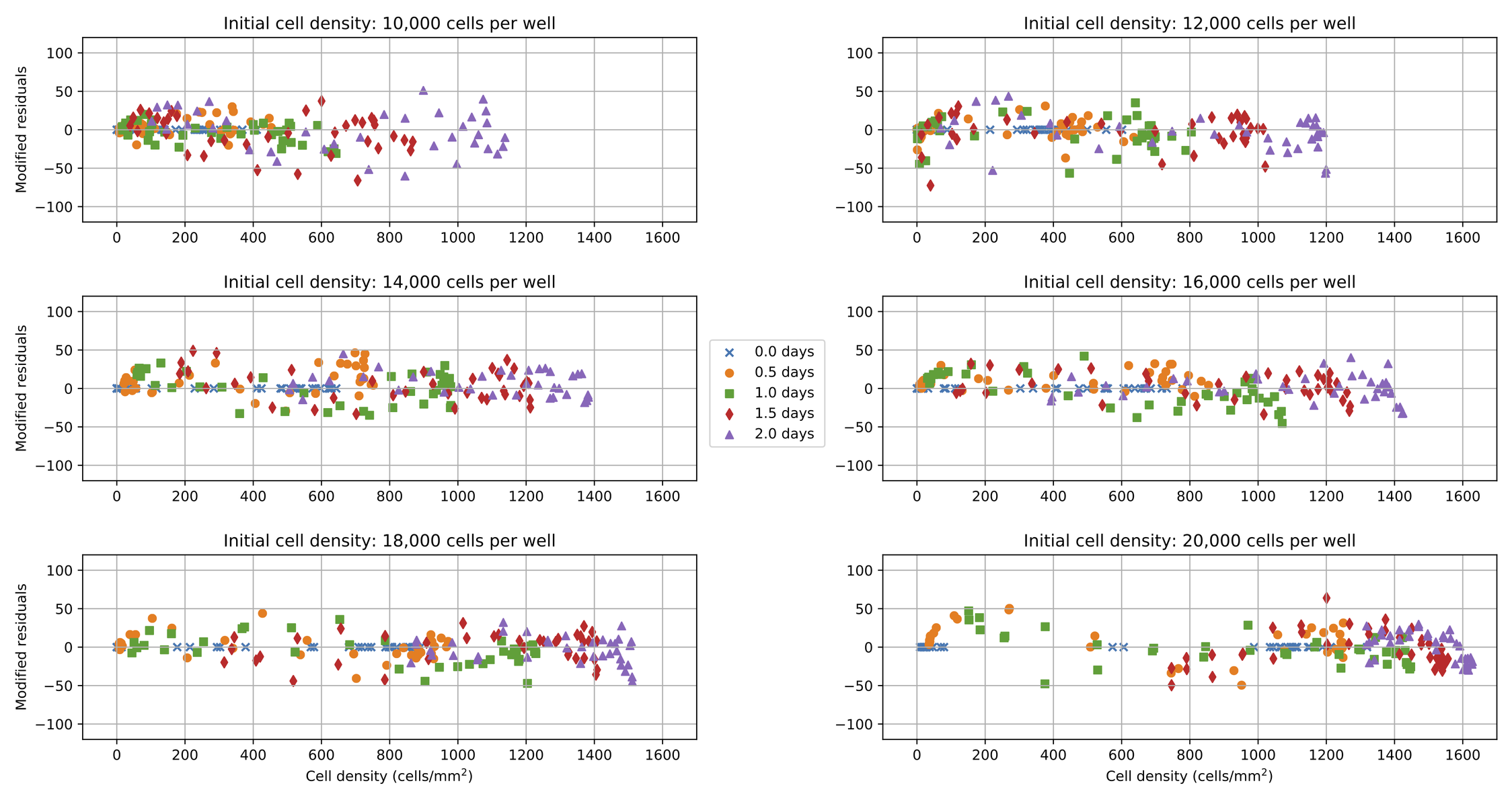

Supplement: S6 Fig — Modified residuals using BINNs with the governing delay-reaction-diffusion PDE in Eq (10). Each subplot corresponds to an experiment with a different initial cell density (i.e. 10,000, 12,000, 14,000, 16,000, 18,000, and 20,000 cells per well). (TIF) [file pcbi.1008462.s006.tif]

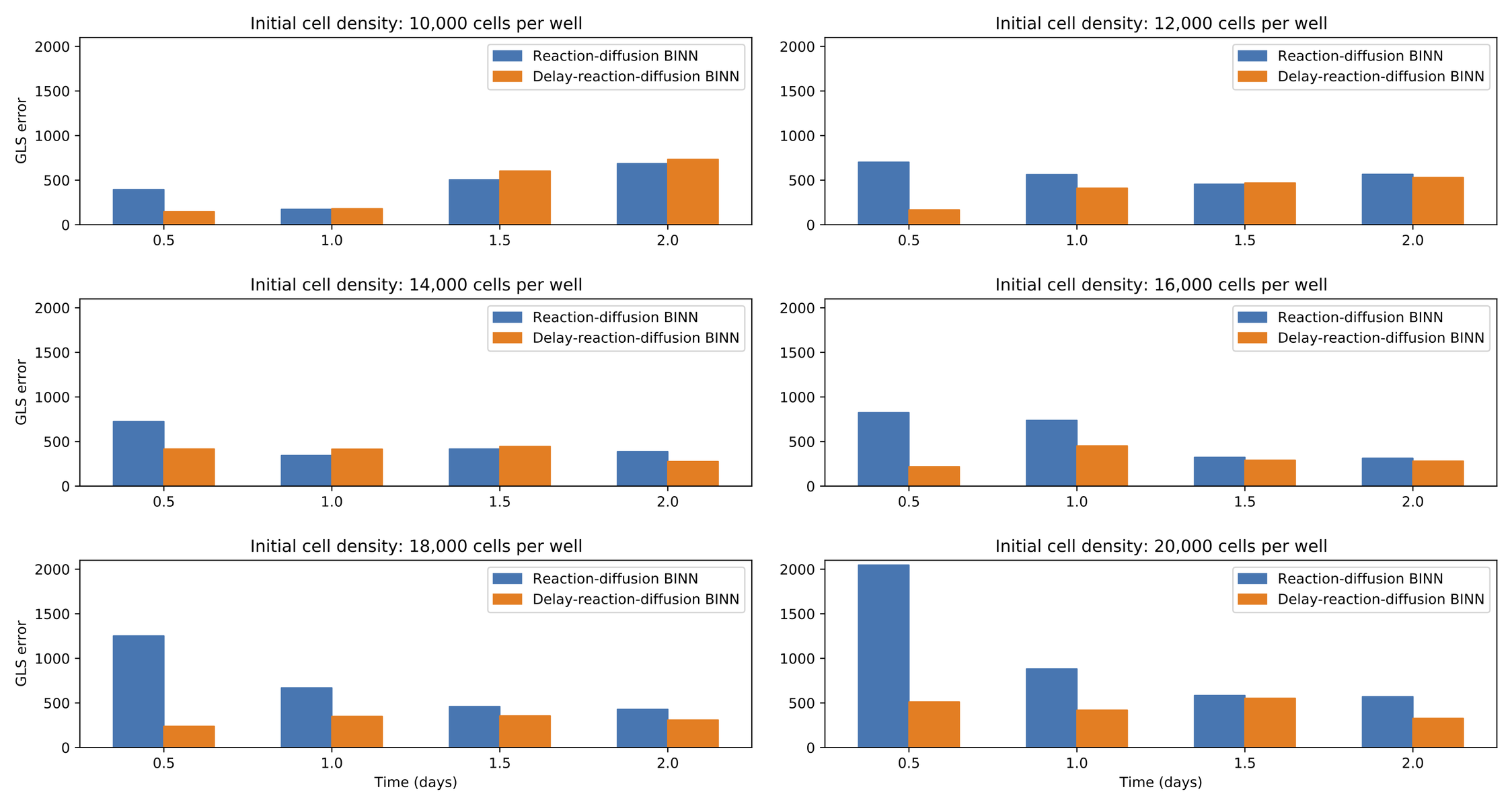

Supplement: S7 Fig — Mean GLS errors between the reaction-diffusion and delay-reaction-diffusion BINNs over the spatial dimension for each time point beyond the initial condition. The initial condition is excluded since the PDE solutions are simulated using the initial condition of the data, meaning that the error at t = 0 is zero. Each subplot corresponds to an experiment with a different initial cell density (i.e. 10,000, 12,000, 14,000, 16,000, 18,000, and 20,000 cells per well). (TIF) [file pcbi.1008462.s007.tif]

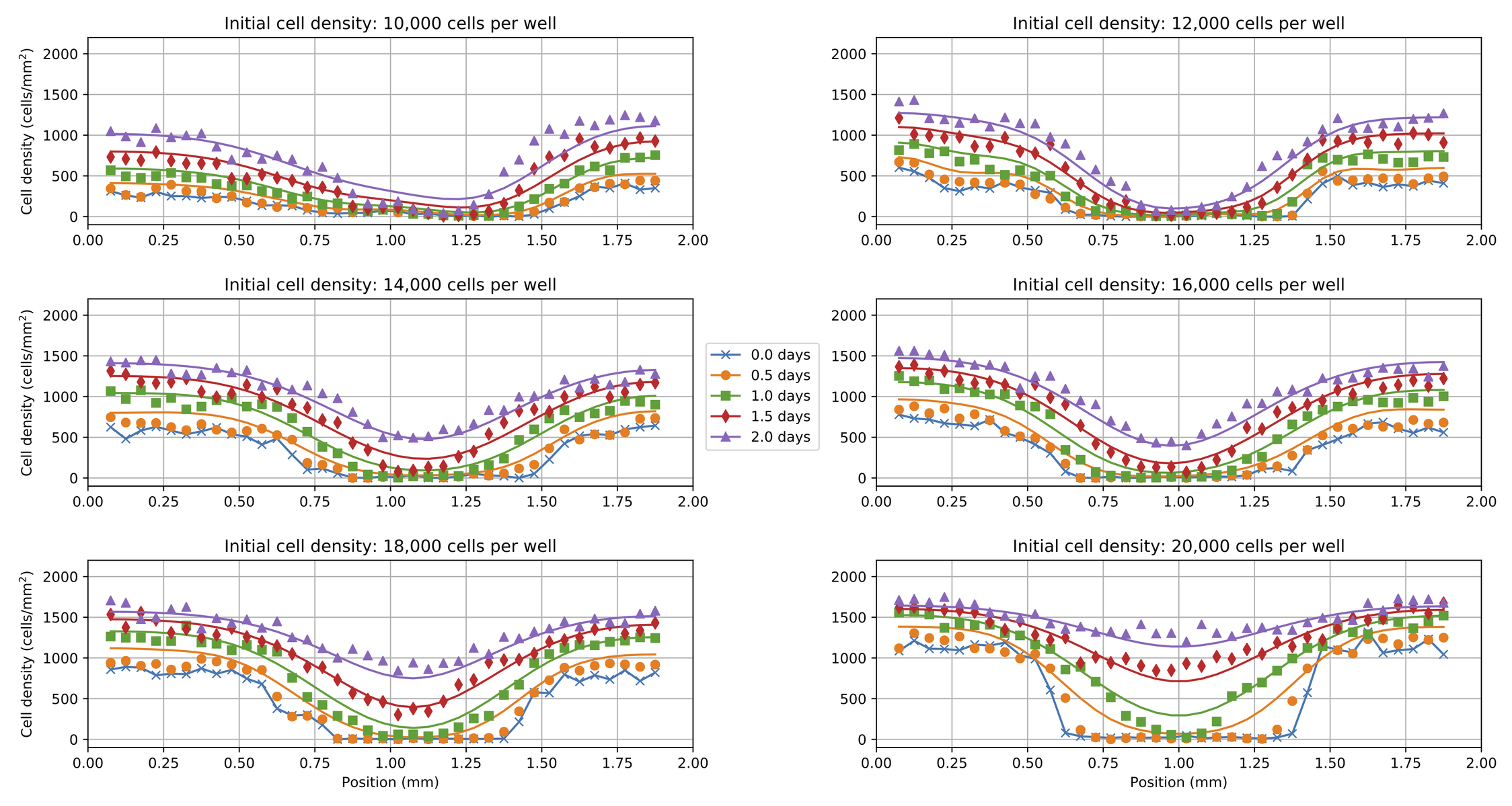

Supplement: S8 Fig — Predicted cell density profiles using the classical FKPP model in Eq (13). Each subplot corresponds to an experiment with a different initial cell density (i.e. 10,000, 12,000, 14,000, 16,000, 18,000, and 20,000 cells per well). Solid lines represent the numerical solution to Eq (13) using the parameters that minimize LGLS in Eq (6). The markers represent the experimental scratch assay data. (TIF) [file pcbi.1008462.s008.tif]

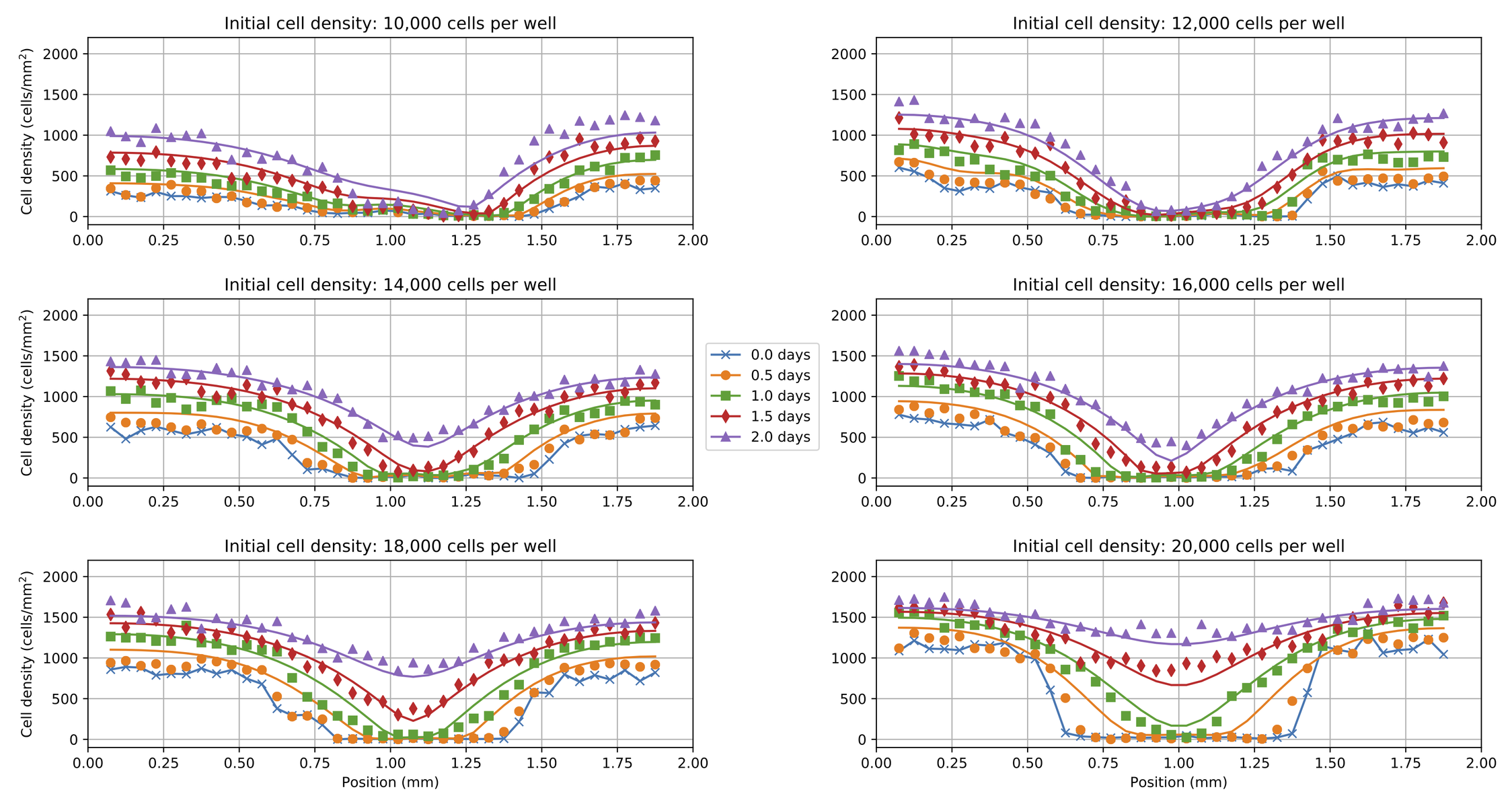

Supplement: S9 Fig — Predicted cell density profiles using the Generalized Porous-FKPP model in Eq (14). Each subplot corresponds to an experiment with a different initial cell density (i.e. 10,000, 12,000, 14,000, 16,000, 18,000, and 20,000 cells per well). Solid lines represent the numerical solution to Eq (14) using the parameters that minimize LGLS in Eq (6). The markers represent the experimental scratch assay data. (TIF) [file pcbi.1008462.s009.tif]

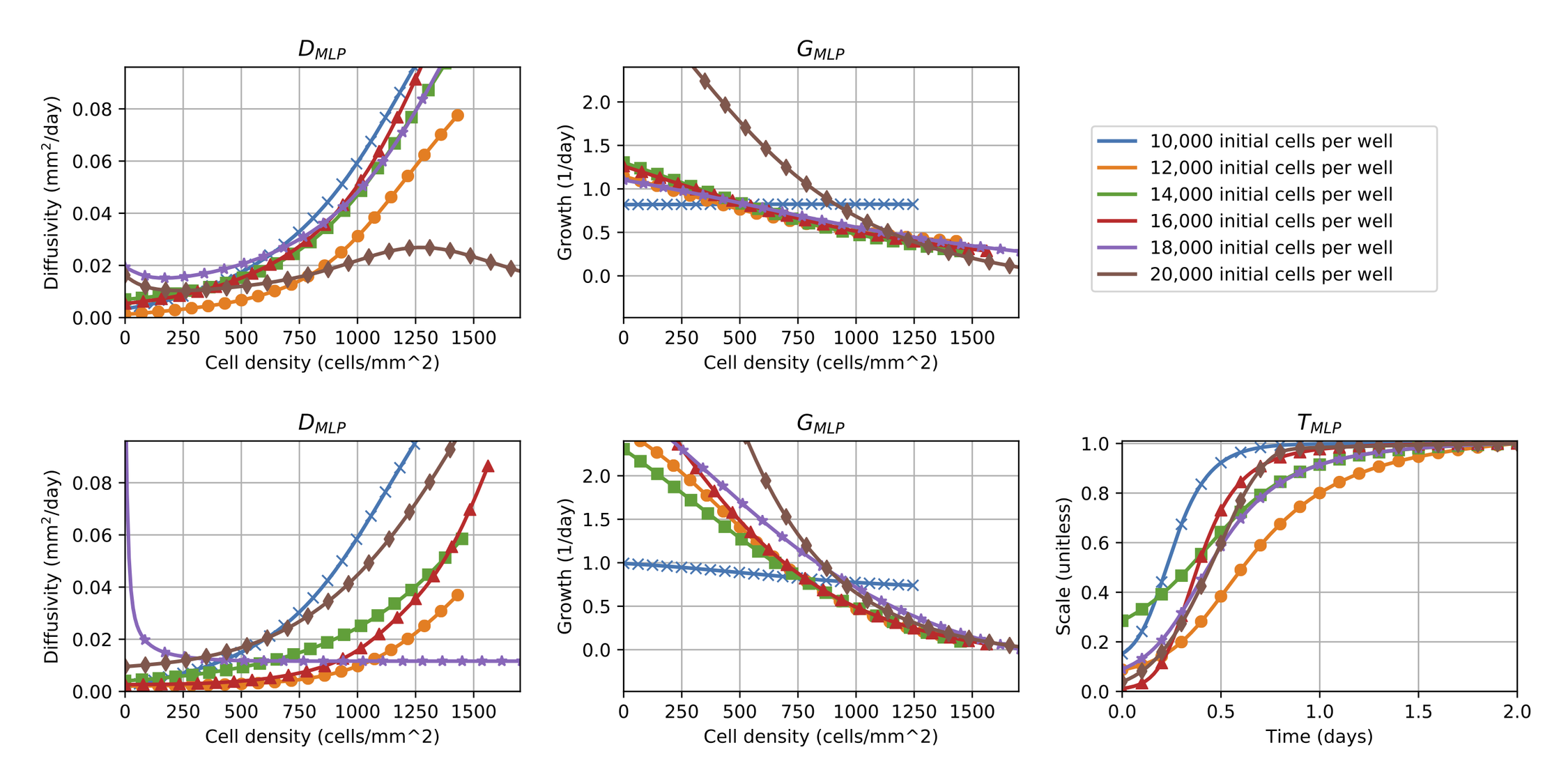

Supplement: S10 Fig — The learned diffusivity DMLP, growth GMLP, and delay TMLP functions extracted from the corresponding BINNs with governing reaction-diffusion PDE in Eq (9) (first row) and delay-reaction-diffusion PDE in Eq (10) (second row). Each line corresponds to an experiment with a different initial cell density (i.e. 10,000, 12,000, 14,000, 16,000, 18,000, and 20,000 cells per well). Note that DMLP and GMLP have different lengths since they are evaluated between the minimum and maximum observed cell densities corresponding to each data set. (TIF) [file pcbi.1008462.s010.tif]

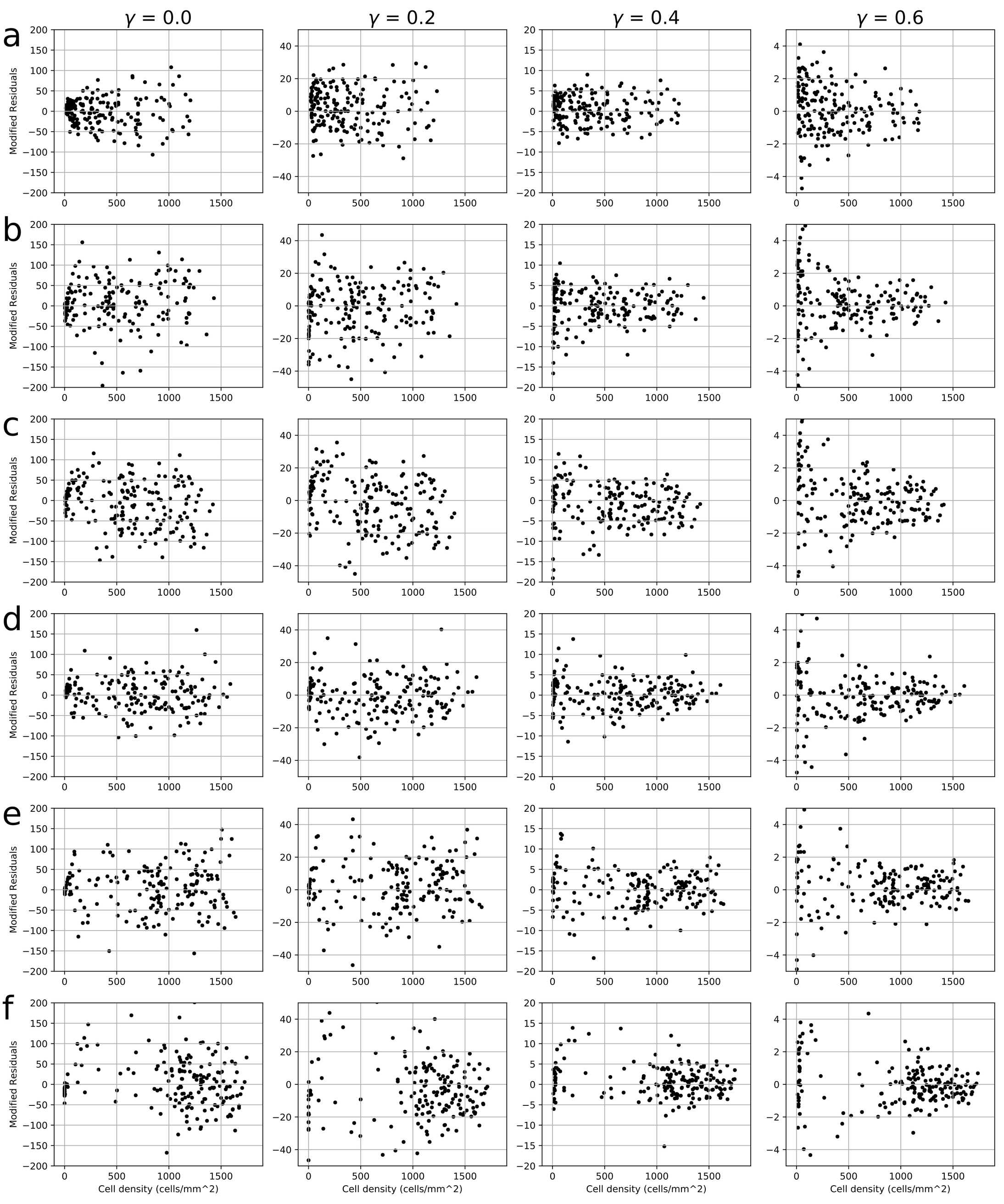

Supplement: S11 Fig — The function-approximating deep neural network uMLP is trained using LGLS for different values of γ across each data set. Each subplot shows the modified residuals (see Eq (6)) as a function of the predicted cell density u. The columns correspond to different levels of proportionality (i.e. γ = 0.0, 0.2, 0.4, 0.6) where γ = 0.0 represents the constant variance (ordinary least squares) case. Each row (a-f) corresponds to an experiment with different initial cell density (i.e. 10,000, 12,000, 14,000, 16,000, 18,000, and 20,000 cells per well). The proportionality constant that results in the most i.i.d. residuals across each data set was chosen to calibrate the statistical error model in Eq (4). (TIF) [file pcbi.1008462.s011.tif]

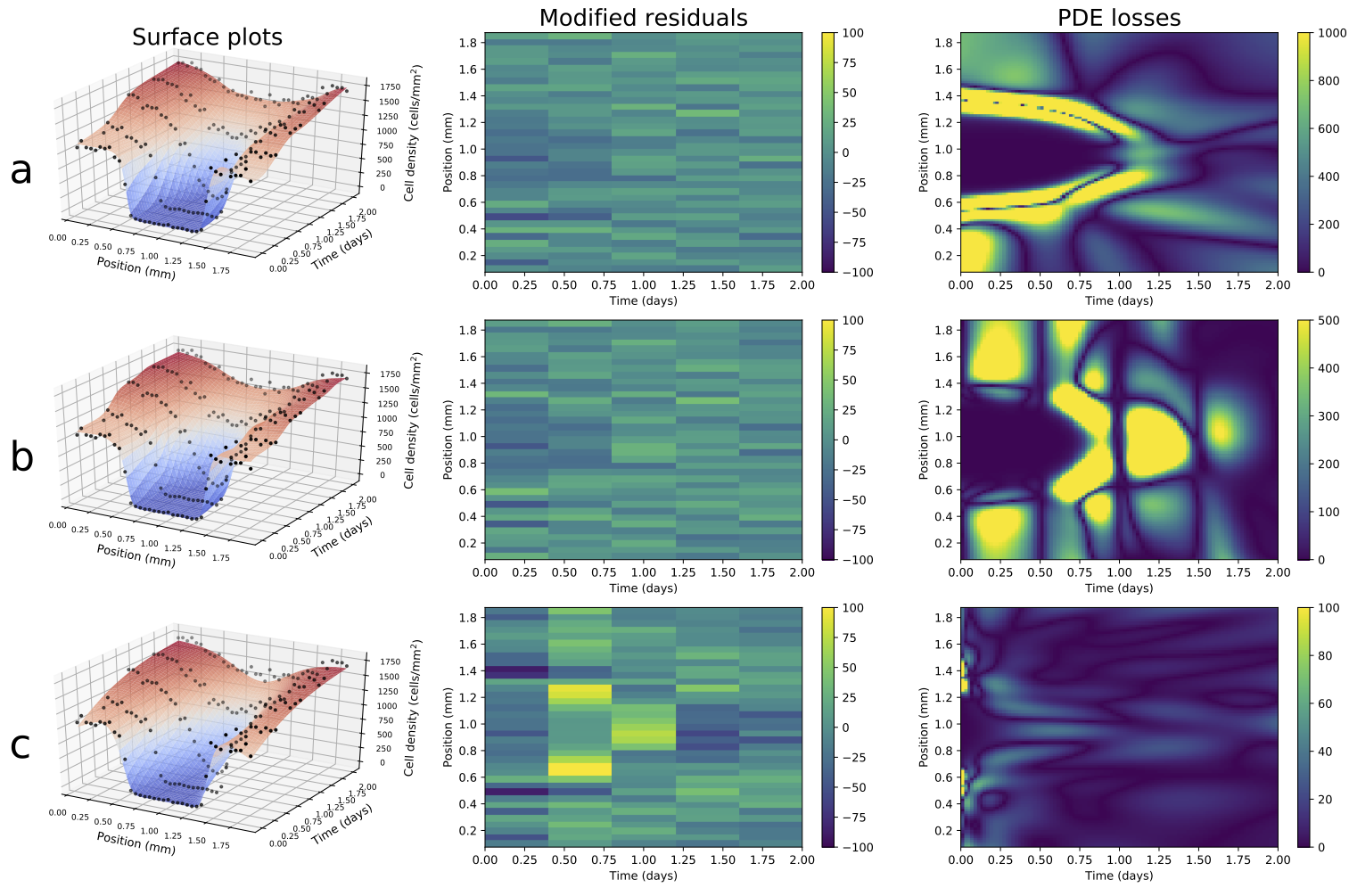

Supplement: S12 Fig — The BINNs framework is trained using LTotal with three ways of including the PDE error term LTotal: (a) no PDE regularization, (b) PDE regularization at the data locations, and (c) PDE regularization at 10,000 randomly sampled points at each training iteration. The first column shows the scratch assay data with initial cell density 20,000 cells per well (black dots) with the corresponding BINNs approximation to the governing PDE uMLP (surface plot). The second column shows heatmaps of the modified residual errors (see Eq (6)) at each data point. The third column shows heatmaps of the PDE errors (see Eq (7)) evaluated on a 100 × 100 meshgrid over the input domain. (TIF) [file pcbi.1008462.s012.tif]

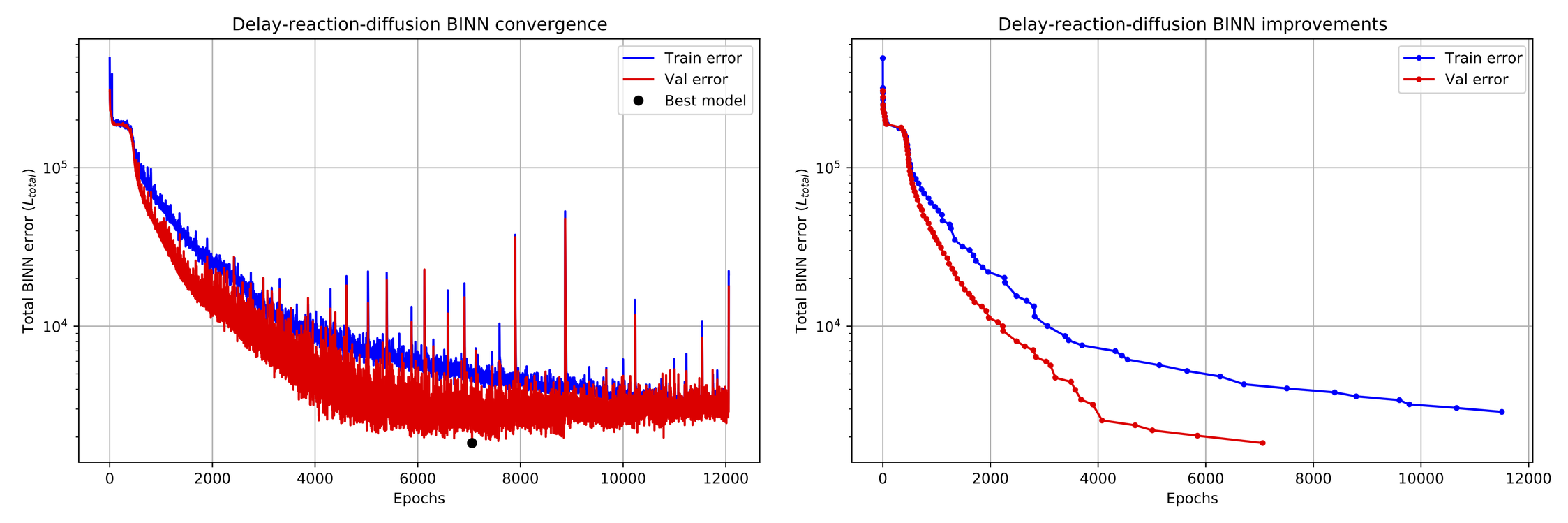

Supplement: S13 Fig — Example convergence and improvement plots from training a delay-reaction-diffusion BINN to the scratch assay data with 20,000 initial cells per well. The left subplot shows the training and validation errors (see Eq (3)) in red and blue, respectively, and the black dot shows where the model achieved the best validation error. Similarly, the right subplot shows the training and validation error but only when the error improved. (TIF) [file pcbi.1008462.s013.tif]
