# Supplementary material for: Biologically-informed neural networks guide mechanistic modeling from sparse experimental data
Source: PLoS Comput Biol. 2020 Dec 1;16(12):e1008462. doi: 10.1371/journal.pcbi.1008462 (PMC7732115; doi:10.1371/journal.pcbi.1008462)
Supplement: S1 Table — Each column corresponds to an experiment with different initial cell density (i.e. 10,000, 12,000, 14,000, 16,000, 18,000, and 20,000 cells per well). (PDF) [file pcbi.1008462.s014.pdf]

	Initial cell density					
Parameter	10,000	12,000	14,000	16,000	18,000	20,000
D ($\mu\text{m}^2/\text{hr}$)	309.7	253.8	681.8	540.9	735.7	978.5
r ($1/\text{hr}$)	0.0437	0.0438	0.0483	0.0490	0.0540	0.0649
