# Supplementary material for: Biologically-informed neural networks guide mechanistic modeling from sparse experimental data
Source: PLoS Comput Biol. 2020 Dec 1;16(12):e1008462. doi: 10.1371/journal.pcbi.1008462 (PMC7732115; doi:10.1371/journal.pcbi.1008462)
Supplement: S2 Table — Each column corresponds to an experiment with different initial cell density (i.e. 10,000, 12,000, 14,000, 16,000, 18,000, and 20,000 cells per well). (PDF) [file pcbi.1008462.s015.pdf]

	Initial cell density					
Parameter	10,000	12,000	14,000	16,000	18,000	20,000
D ($\mu m^2/hr$)	1851.8	465.0	2993.4	2371.0	2377.8	2017.1
m (unitless)	0.9704	0.3001	0.9923	0.9879	0.8265	0.6235
r ($1/hr$)	0.0435	0.0436	0.0481	0.0484	0.0526	0.0639
